# Use of Repetitive Sequences for Molecular and Cytogenetic Characterization of *Avena* Species from Portugal

**DOI:** 10.3390/ijms17020203

**Published:** 2016-02-04

**Authors:** Diana Tomás, Joana Rodrigues, Ana Varela, Maria Manuela Veloso, Wanda Viegas, Manuela Silva

**Affiliations:** 1 Linking Landscape, Environment, Agriculture and Food (LEAF), Instituto Superior de Agronomia, Universidade de Lisboa, Tapada da Ajuda, 1349-017 Lisboa, Portugal; dianarstomas@isa.ulisboa.pt (D.T.); joana.jr.rodrigues@gmail.com (J.R.); amsvg_89@hotmail.com (A.V.); mveloso.inrb@gmail.com (M.M.V.); wandaviegas@isa.ulisboa.pt (W.V.); 2 Instituto Nacional de Investigação Agrária e Veterinária, Quinta do Marquês, 2784-505 Oeiras, Portugal

**Keywords:** *Avena* Portuguese lines, repetitive sequences, genomic diversity, molecular markers

## Abstract

Genomic diversity of Portuguese accessions of *Avena* species—diploid *A. strigosa* and hexaploids *A. sativa* and *A. sterilis*—was evaluated through molecular and cytological analysis of 45S rDNA, and other repetitive sequences previously studied in cereal species—rye subtelomeric sequence (pSc200) and cereal centromeric sequence (CCS1). Additionally, retrotransposons and microsatellites targeting methodologies—IRAP (inter-retrotransposon amplified polymorphism) and REMAP (retrotransposon-microsatellite amplified polymorphism)—were performed. A very high homology was detected for ribosomal internal transcribed sequences (ITS1 and ITS2) between the species analyzed, although nucleolar organizing regions (NOR) fluorescent *in situ* hybridization (FISH) analysis revealed distinct number of *Nor loci* between diploid and hexaploid species. Moreover, morphological diversity, evidenced by FISH signals with different sizes, was observed between distinct accessions within each species. pSc200 sequences were for the first time isolated from *Avena* species but proven to be highly similar in all genotypes analyzed. The use of primers designed for CCS1 unraveled a sequence homologous to the Ty3/gypsy retrotransposon *Cereba*, that was mapped to centromeric regions of diploid and hexaploid species, being however restricted to the more related A and D haplomes. Retrotransposon-based methodologies disclosed species- and accessions-specific bands essential for the accurate discrimination of all genotypes studied. Centromeric, IRAP and REMAP profiles therefore allowed accurate assessment of inter and intraspecific variability, demonstrating the potential of these molecular markers on future oat breeding programs.

## 1. Introduction

*Avena* genus (2*n* = 2*x* = 14) comprises diploid (A and C genomes), tetraploid (AB and AC genomes) and hexaploid (ACD) species. Hexaploid species appear to have arisen by two rounds of hybridization followed by chromosome duplication whereas tetraploid species with AB genomes were suggested to result from an unrelated event involving an A diploid species autopolyploidization (for a review see [1]). Structurally different A and C genomes have variants and B and D genomes are considered to be derived from an A genome [1]. Only four of 27 *Avena* species [2] are cultivated and used for food, feed or forage, namely: diploid *A. strigosa* (A), tetraploid *A. abyssinica* (AB) and hexaploids *A. byzantine* and *A. sativa* (ACD) [1]. *A. strigosa* and *A. sativa* are the major *Avena* crops in Portugal, which is considered the center of diversity of the former one [1]. On the other hand, the most relevant *Avena* wild species in Portugal is *A. sterilis* [3] with a high potential in oat breeding programs due to important agronomic traits increased grain yield and protein content and resistance to abiotic and biotic stresses [4]. In fact, natural hybrids between *A. sativa* and *A. sterilis* occur frequently and are widespread in Portugal [3].

Several molecular markers such as Restriction Fragment Length Polymorphism (RFLP), Amplified Fragment Length Polymorphism (AFLP), microsatellites and rDNA sequences were previously used to survey genomic diversity in *Avena* genus [5,6,7]. *Avena* genus phylogenetic correlations have been evaluated through the analysis of 45S rDNA Internal Transcribed Spacers (ITS1 and ITS2) of species with distinct genome composition [8,9,10,11]. On the other hand, RFLP, microsatellites and Inter-Simple Sequence Repeat (ISSR) were used to geographically cluster accessions of *A. strigosa* [12], *A. sativa* [13,14] and *A. sterilis* [15,16]. Inter-retrotransposon amplified polymorphism (IRAP) and retrotransposon-microsatellite amplified polymorphism (REMAP) methodologies were initially designed to identify different barley (*H. vulgare*) cultivars [17]. Such PCR-based techniques target retrotransposons long terminal repeat (LTR) sequences relevant in genome evolution and speciation due to their mobile nature [18] and microsatellites *loci* that are preferentially associated with retrotransposons in cereals [19]. Those methodologies were recently used for genomic characterization of monocotyledonous crop species such as wheat (*T. aestivum*), rye (*S. cereale*), triticale (x*Triticosecale*) (reviewed in [20]), rice (*O. sativa*) [21], maize (*Z. mays*) [22], cocoyam (*Xanthosoma sagittifolium*) and taro (*Colocasia esulenta*) [23]. REMAP methodologies were also previously used as markers associated with *A. sativa* agronomic traits [24] as well as to assess *A. sativa*, *A. sterilis* and *A. fatua* evolutionary relationships [25]. Molecular characterization of such dispersed sequences as well as other repetitive sequences is therefore important to better evaluate *Avena* landraces genomic diversity. Rye subtelomeric pSc200 sequences for instance were already relevant to disclose differences between cereal genera such as *Secale*, *Triticum* and *Hordeum* [26] although not yet studied in *Avena*. A similar situation occurs with the highly conserved cereal centromeric sequence (CCS1) firstly isolated from *Brachypodium sylvaticum* [27] and already used to characterize several species as barley (*Hordeum vulgare*), wheat (*T. aestivum*), rye (*S. cereale*), maize (*Zea mays*) and rice (*Oryza sativa*) either through Southern blot, PCR analysis or fluorescent *in situ* hybridization [27,28].

In this work, the characterization of distinct Portuguese accessions of *A. strigosa*, *A. sativa* and *A. sterilis* was performed through the analysis of distinct repetitive genome fractions, including *Nor loci* (ITS1 and ITS2) as well as sequences previously mapped in other cereals at centromeric (CCS1) and subtelomeric (pSc200) chromosome domains. Sequences dispersed throughout the genome such as retrotransposons and microsatellites were also analyzed through IRAP and REMAP methodologies. Besides interspecific variability, the results obtained also disclosed intraspecific genetic diversity in Portuguese *Avena* species allowing the establishment of molecular markers that may be useful to reliably identify valuable genotypes.

## 2. Results

To assess *Avena* diversity we analyzed distinct repetitive sequences such as 45S rDNA ITS1 and ITS2 and sequences previously mapped on subtelomeric (pSc200) and centromeric (CCS1) domains in other cereal species. Retrotransposons and microsatellites flancking sequences were also analyzed through IRAP (Inter Retrotransposons Amplified Polymorphism) and REMAP (Retrotransposons Microsatellite Amplified Polymorphism) methodologies. All molecular markers used revealed unique and consistent banding profiles in at least three distinct individuals of each genotype of *A. strigosa*, *A. sativa* and *A. sterilis* demonstrating the inexistence of intravarietal variability. The total number of bands per molecular marker used and genotype analyzed are summarized in Table 1. 45S rDNA ITS1 and ITS2 and pSc200 amplification yielded a single band in all *Avena* genotypes that was further isolated, purified and cloned for sequences analysis (Accession Numbers in Table S1). Centromeric sequences, IRAP and REMAP yielded distinct banding profiles for each genotype which were further compared using NTSYSpc software (Numerical Taxonomy and Multivariate Analysis System).

**Table 1:** Number of bands per banding profile obtained in each PCR experiment.

Accession	45S ITS1	45S ITS2	pSc200	CCS1	IRAP	REMAP
*A. strigosa* “Madeira Island”	1	1	1	5	9	15
*A. strigosa* “Elvas”	1	1	1	5	9	14
*A. sativa* “Kyto”	1	1	1	6	10	14
*A. sativa* “Madeira Island”	1	1	1	7	8	9
*A. sativa* “S. Eulália”	1	1	1	5	11	13
*A. sterilis*	1	1	1	4	10	9

ITS1 and ITS2: 45S rDNA internal transcribed spacers; pSc200: rye subtelomeric sequence (Accession Number Z50039); CCS1: cereal centromeric sequences (Accession Number, U52217), IRAP Nikita and REMAP Nikita (CA)9G.

### 2.1. 45S rDNA Internal Transcribed Sequences Similarity and Nor Loci Diversity

Primers targeting 45S rDNA sequences [29] produced single bands with the expected sizes (~300 and ~380 bp, respectively) in all species/accessions analyzed as well as in pTa71 plasmid used as control. ITS1 and ITS2 sequences included in those amplified products present 219 and 216 bp, respectively, as already described for other *Avena* species [11]. Moreover, ITS sequence analysis using original parameters of Clustal W2 [30] revealed a high level of similarity—between 98% and 100% for ITS1 and between 99% and 100% for ITS2. ITS1 sequences alignment presented six polymorphic nucleotides and 100% identity was detected between *A. sativa* “S. Eulália” and *A. sterilis.* In ITS2 sequences only one polymorphic nucleotide was detected between *A. strigosa* and *A. sativa* “Madeira Island” from *A. sativa* Kyto, S. Eulália and *A. sterilis.*

**Figure 1:**
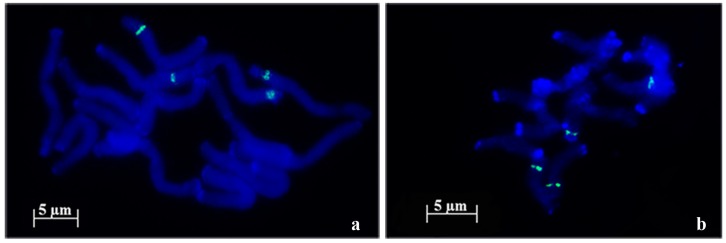
45S rDNA fluorescent *in situ* hybridization (green signals) in metaphase cells of diploid *A. strigosa*. In the landrace from Madeira Island (**a**) two pairs of nucleolar organizing regions (NORs) with distinct sizes are observed and in the landrace from Elvas; (**b**) two pairs of NORs with similar sizes are detected.

**Figure 2:**
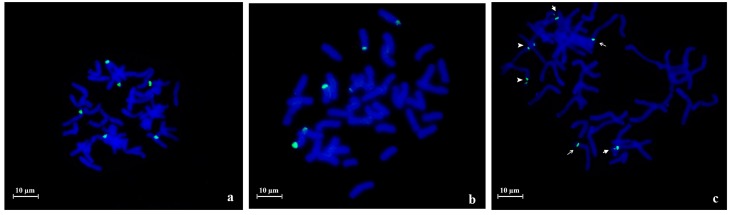
45S rDNA fluorescent *in situ* hybridization (green signals) in metaphase cells of *Avena* hexaploid species. Three pairs of NORs are detected both in *A. sativa* as in *A. sterilis*. (**a**) *A. sativa* “S. Eulália” presenting NORs with similar sizes; (**b**) *A. sativa* from Madeira Island showing one large pair of NORs, one medium size pair and one heteromorphic pair (one medium size and one small); (**c**) *A. sterilis* showing one NOR pair with one signal (long line arrow); a second pair composed by two domains with different sizes (short line arrow); and a third one with two domains with similar sizes (arrow head).

*Nor loci* evaluation performed in root tip cells of *A. strigosa* landraces revealed four FISH positive NORs using pTa71 probe (Figure 1), presenting however some intraspecific morphological variability. In fact, in “Madeira Island” landrace two pairs of NORs with distinct sizes are observed (Figure 2a) and in “Elvas” landrace the four NORs exhibit similar sizes (Figure 2b).

In hexaploid species *A. sativa* and *A. sterilis*, 45S rDNA FISH revealed three pair of signals (Figure 2). However, in *A. sativa* distinct landraces revealed *Nor loci* diversity in relation to NORs size. While in “S. Eulália” all three NOR pairs exhibit similar sizes (Figure 2a), “Madeira Island” landrace presented FISH signals with different sizes corresponding to one pair of large NORs, one pair of medium size NORs and one heteromorphic pair composed by one medium size and one minor NOR (Figure 2b). Furthermore, in *A. sterilis* prometaphase cells with distended chromosomes, the three NOR pairs are distinguished through their different topological organization since one pair presents a single FISH domain whereas the other two pairs show more than one labeled domain intercalated by an unlabeled region that corresponds most probably to transcriptionally active uncondensed rDNA units. In one pair of NORs the two domains have similar dimensions, contrasting with the other pair where both domains have different dimensions (Figure 2c). The numbers of rDNA FISH signals and their described organization patterns were observed in at least 10 metaphase or prometaphase cells and are summarized in Table 2.

**Table 2:** rDNA FISH signals.

Species	Accession	Number of NORs	Description
*A. strigosa*	“Madeira”	4	1 large pair and 1 small pair
	“Elvas”	4	2 pairs with similar size
*A. sativa*	“Madeira Island”	6	1 large pair, 1 medium pair and 1 heteromorphic pair (1 medium and 1 small)
	“S. Eulália”	6	3 pairs with similar sizes
*A. sterilis*	–	6	1 pair with 1 signal, 1 pair with 2 signals with similar sizes and 1 pair with 2 signals with different sizes

### 2.2. Novel Repetitive Sequences Were Identified Contributing for Avena Accessions Discrimination

**Figure 3:**
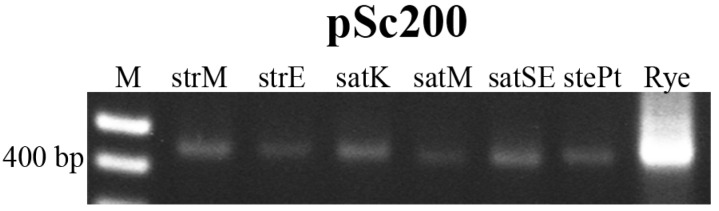
PCR amplification products obtained using pSc200 primers. M: molecular marker 1 Kb+; strM—*A. strigosa* from Madeira Island; strE—*A. strigosa* from Elvas; satK—*A. sativa* “Kyto”; satM—*A. sativa* from Madeira Island; satSE—*A. sativa* “S. Eulália”; stePt—*A. sterilis* Pt; Rye—*S. cereale*.

Genomic diversity evaluation in *Avena* was extended through the analysis of repetitive sequences previously described in subtelomeric and centromeric domains in other cereal species [26]. The amplification products obtained with primers designed to pSc200 sequence are presented in Figure 3. A 444 bp fragment is obtained, as a result of the amplification between the forward and reverse primers in subsequent units, as already described in [31], including in all species/accessions analyzed a single complete unit with 379 bp as described in rye [32], and where a significantly more intense band is observed when rye genomic DNA is used as control. Those bands were isolated, cloned and sequenced (Accession Numbers in Table S1) and their BLAST (Basic Local Alignment Search Tool) analysis in NCBI database revealed between 99%–100% similarity with rye subtelomeric sequence (Accession Number Z50039, [32] and 90% homology with a *A. sativa* sequence (Genbank Accession Number DQ481575, unpublished data). Moreover, the homology level between pSc200-like sequences isolated from the distinct *Avena* genotypes analyzed (alignment in Figure S1) is extremely high (~99%).

FISH analysis on the *Avena* genotypes studied was performed using pSc200 probe and rDNA pTa71 probe as positive control. Contrary to the rDNA probe that was detected in all metaphase spreads, the pSc200 probe was not detected in any *Avena* accession.

**Figure 4:**
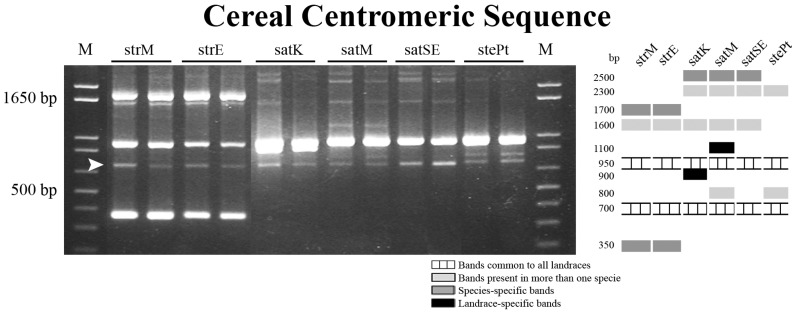
PCR amplification products obtained using CCS1 primers and correspondent schematic representation. M: molecular marker 1 Kb+; strM—*A. strigosa* from Madeira Island; strE—*A. strigosa* from Elvas; satK—*A. sativa* “Kyto”; satM—*A. sativa* from Madeira Island; satSE—*A. sativa* “S. Eulália”; stePt—*A. sterilis* Pt. Arrowhead indicates the *Avena* characteristic band that was amplified from all genotypes, isolated and sequenced.

Another repetitive sequence hitherto mapped in cereal centromeric regions (CCS1) was studied in *Avena* through PCR amplification and FISH evaluation. The PCR profiles obtained are distinct in the three *Avena* species analyzed and contain bands ranging in size from 350 to 2500 bp (schematic representation in Figure 4). Two bands are common to all genotypes analyzed, one with a size identical (950 bp) to the published maize centromeric sequence [28] and a novel one with 700 bp. Both *A. strigosa* landraces exhibit identical profiles, containing besides the two common bands, three other bands, two *A. strigosa*-specific (~380 and ~1700 bp) and one common to *A. sativa* (~1650 bp). *A. sativa* and *A. sterilis* banding profiles present one band characteristic of *Avena* hexaploid species with ~2300 bp as well as a *A. sativa*-specific band with ~2500 bp, which distinguishes the hexaploid species studied. Also, *A. sativa* landraces present very similar profiles composed of five *A. sativa* common bands previously described, plus “Kyto” or “Madeira Island” specific bands (~900 and ~1100 bp, respectively). In *A. sterilis*, besides the two *Avena* characteristic bands and the hexaploid-specific band, a fourth band with ~800 bp is observed similar to the one present in *A. sativa* landrace from Madeira Island.

The *Avena* characteristic novel band with ~700 bp was isolated, cloned and sequenced (Accession Numbers in Table S1) and the similarity values between genotypes range from 89% to 98% (Alignment in Figure S2). No similarity with other *Avena* centromeric sequences already published in Genbank was found. However, it yielded significant alignment (~72%) with retrotransposon-related centromeric sequences of *Triticum* (Acession Number HF541873.1) [33], *Secale* (Accession Number JQ963524.1) [34] and *Hordeum* (Accession Number AY040833.1) [35], targeting the long terminal region of the mobile element Ty3/gypsy retrotransposon *Cereba* firstly characterized in *H. vulgare* [35].

**Figure 5:**
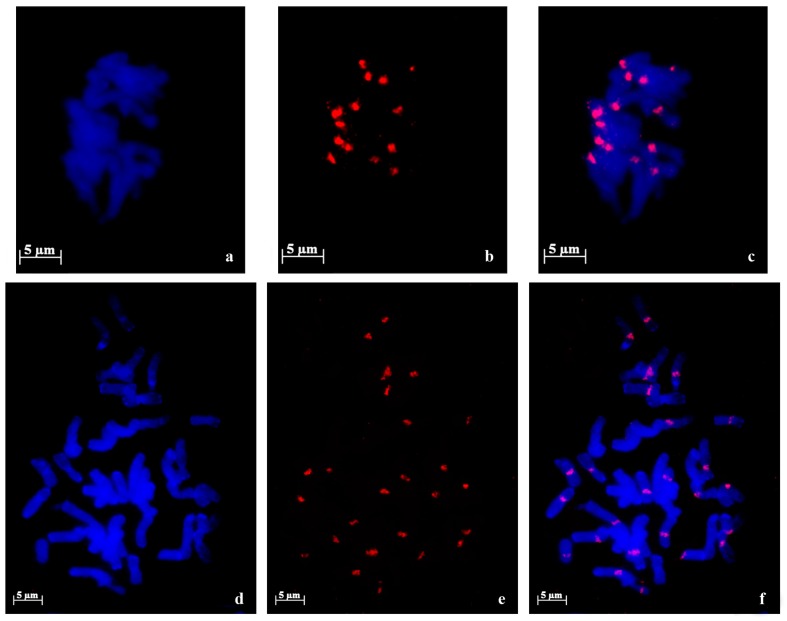
Centromeric sequence fluorescent *in situ* hybridization (red signals) in metaphase cells. Metaphase cells of *A. strigosa* (**a**–**c**); and *A. sterilis* (**d**–**f**) with DAPI staining (**a**,**d**), FISH with centromeric probe (Accession Number KM948610) (**b**,**e**) and merge of both fluorescent signals (**c**,**f**).

The *Avena* novel sequence amplified from *A. sativa* was labeled with biotin-dUTP and used as a probe for FISH evaluation in spreads of the species studied (Figure 5). In *A. strigosa* metaphase cells all centromeric domains are FISH-positive using that centromeric probe (Figure 5a–c) and although hexaploid species also revealed discrete signals, only 28 from the 42 chromosomes were FISH labeled (Figure 5d–f). Thus, 14 chromosomes, probably composing one *Avena* haplome, do not show centromeric labeling.

Conclusively, the comparative analysis of amplification products obtained with CCS1 primers allows a clear discrimination of the three *Avena* species in the study. Moreover, *A. sativa* accessions exhibit distinct banding profiles since two landrace-specific bands were identified. The two *A. strigosa* accessions are not distinguishable, thus other molecular markers were further used to seek for a complete discrimination of all *Avena* genotypes analyzed.

### 2.3. Retrotransposon and Microsatellite Flanking Sequences Untangled Avena Accessions Discrimination

**Figure 6:**
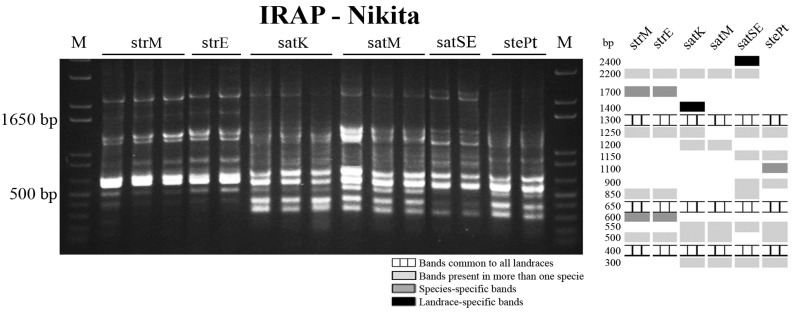
Inter-retrotransposon amplified polymorphism (IRAP) banding profiles obtained with primer for Nikita retrotransposon long terminal repeat (LTR) and correspondent schematic representation. M: molecular marker 1 Kb+; strM—*A. strigosa* from Madeira Island; strE—*A. strigosa* from Elvas; satK—*A. sativa* “Kyto”; satM—*A. sativa* from Madeira Island; satSE—*A. sativa* “S. Eulália”; stePt—*A. sterilis* Pt.

The amplification of sequences flanking *Nikita* retrotransposon LTRs is presented in Figure 6. Banding profiles analysis allowed the identification of three bands common to all genotypes with ~400, ~650 and ~1300 bp (schematic representation in Figure 6). Both *A. strigosa* accessions showed identical banding patterns with two species-specific bands (~600 and ~1700 bp) besides the three common ones. In *A. sativa* two landrace-specific bands were detected in “Kyto” and “S. Eulália” (~1400 and ~2400 bp, respectively) and *A. sativa* from Madeira Island lacks the ~1250 bp band present in all other genotypes analyzed. Finally, the *A. sterilis* Pt banding profile includes a specific band with ~1100 bp (Figure 6). Thus, using the IRAP marker was not yet possible to distinguish the two *A. strigosa* landraces.

**Figure 7:**
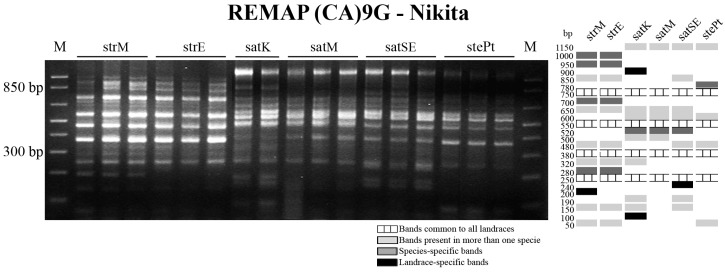
REMAP banding profiles obtained with primer for Nikita retrotransposon LTR and (CA)9G microsatellite anchored primer and correspondent schematic representation. M: molecular marker 1 Kb+; strM—*A. strigosa* from Madeira Island; strE—*A. strigosa* from Elvas; satK—*A. sativa* “Kyto”; satM—*A. sativa* from Madeira Island; satSE—*A. sativa* “S. Eulália”; stePt—*A. sterilis* Pt.

REMAP analysis performed using the same retrotransposon primer combined with the (CA)9G microsatellite anchored primer yielded a banding profile (Figure 7 with schematic representation) that includes four bands common to all *Avena* genotypes analyzed with ~250, ~380, ~550 and ~750 bp, respectively. *A. strigosa* banding profile contains also four bands characteristic of this species (~280, ~700, ~950 and ~1000 bp) and the landrace from Madeira Island presents a specific one (~200 bp). Hexaploid species profiles, besides containing the bands common to all genotypes, include two common bands (with ~600 and ~1150 bp). Furthermore, both in *A. sativa* and *A. sterilis* two species-specific bands with ~520 and ~780 bp were identified. Also, two landrace-specific bands were also identified in *A. sativa* “Kyto” (~100 and ~900 bp) and one in “S. Eulália” (~240 bp). Thus, the banding patterns obtained with this REMAP *Nikita*/(CA)9G marker allowed the clear distinction between all *Avena* genotypes evaluated.

**Figure 8:**
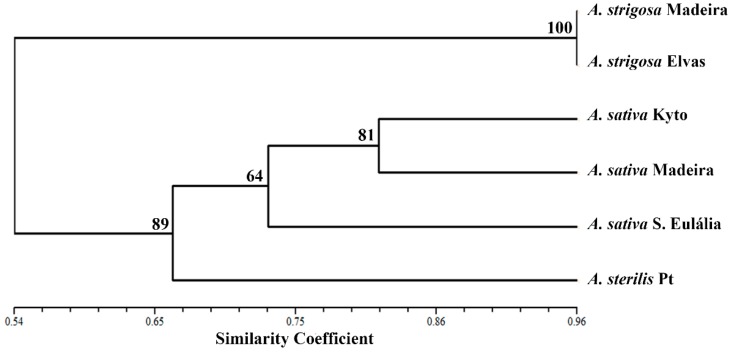
Dendogram based on concatenated centromeric, IRAP and REMAP banding profiles. DICE similarity index [36] and UPGMA clustering mode were used. Cophenetic correlation coefficient of 0.996.

*Avena* species/accessions genomic diversity evaluation was performed using Cluster analysis of IRAP, REMAP and centromeric banding profiles concatenated by NTSYSpc software, and the dendrogram produced is presented in Figure 8. As expected, *A. strigosa* landraces revealed to be sister taxa forming a monophyletic group and the hexaploid genotypes analyzed are clustered in another group. Moreover, the three *A. sativa* genotypes evaluated are gathered in a sub-group with a similarity coefficient of ~0.74. *A. sativa* “Kyto” and *A. sativa* from Madeira Island present higher similarity (with similarity coefficient ~0.82). Concordantly, the bootstrap value of the *A. sativa* node is the lowest one demonstrating the great similarity between the *A. sativa* genotypes analyzed. *A. sterilis* integrates the hexaploid *Avena* species branch although clearly separated from *A. sativa* cluster with the lowest similarity coefficient (0.67).

## 3. Discussion

In this work we aimed to portray the genomic diversity of *Avena* species cultivated in Portugal–*A. strigosa* and *A. sativa*, as well as the most representative *Avena* wild relative*—A. sterilis*, through molecular and cytogenetic analysis of distinct coding and non-coding repetitive sequences. The analysis of 45S rDNA internal transcribed sequences revealed high similarity between *A. strigosa*, *A. sativa* and *A. sterilis—*98% and 100% in ITS1 and ITS2, respectively, corroborating the great homology previously reported for these sequences in several phylogenetic studies of *Avena* genus [8,10,11]. However, 45S rDNA assessment through fluorescent *in situ* hybridization (FISH) revealed some *Nor loci* topological diversity. In both *A. strigosa* landraces, two pairs of NORs were identified, as formerly described for other *A. strigosa* lines [37,38], although only one accession presents pairs of NORs with distinct sizes as previously described in *A. strigosa* accession PI 258729 [37]. In *A. sativa* six FISH signals were observed in accordance with the number of *Nor loci* previously described [37,39,40,41,42]. Though, while in “S. Eulália” all NORs exhibit similar sizes as previously reported in other *A. sativa* lines [43,44], *A. sativa* landrace from Madeira Island shows NOR pairs with distinct dimensions as well as one heteromorphic *Nor* pair. In *A. sterilis* six 45S rDNA FISH signals were also detected as formerly reported for this species [37,39,40,41]. Moreover, distinct physical organization patterns for each pair of NORs were also described, similar to the organization inferred for *A. byzantina* NORs as revealed in C-banded chromosomes [42]. Thus, in contrast to the constancy of rDNA molecular analysis, *Nor loci* cytological evaluation allowed the identification of distinct ribosomal chromatin organization patterns.

Portuguese *Avena* species diversity evaluation was also studied through the analysis of other repetitive sequences previously assessed in cereals, such as pSc200 sequence—a major family of sequences isolated from rye and mapped in subtelomeric domains [32,45]. In this work we amplified in *Avena* a 444 bp fragment with primers previously used in rye and wheat lines with rye chromatin introgression [31] and sequence analysis revealed a high level of similarity (99%–100%) with rye original pSc200 sequence (Accession Number Z50039) [26]. pSc200 sequences were also detected by Southern blot and PCR as well as by FISH analysis in other Triticeae species from the genera *Secale*, *Agropyron* and *Dasypyrum* [26]. However, none of the *Avena* genotypes studied presented any detectable cytological signal, probably due to the reduced number of pSc200-like copies, substantiating the idea that the copy number of this repetitive sequences is extremely variable in distinct cereal *taxa*, as proposed by [26].

Contrasting with the molecular analysis of ITS and pSc200 sequences, cereal centromeric sequence (CCS1) strongly contributed to assess *Avena* genotypes diversity, as previously used for other cereals such as *H. vulgare*, *S. cereale* and *T. aestivum* [27,28]. PCR with CCS1 primers yielded consistent banding profiles that strengthen our analysis of *Avena* genotypes allowing the discrimination of most genotypes analyzed with the exception of the two *A. strigosa* landraces. CCS1 banding profiles obtained include a novel *Avena* band with ~700 bp with high similarity in all accessions studied. The BLAST analysis of this sequence targeted retrotransposon-related centromeric sequences isolated from cereals [33,34], showing a high similarity with the LTR of *Cereba* retrotransposon firstly identified in *Hordeum vulgare* [35], supporting therefore previous works on retrotransposon-like sequences richness in grasses centromeres [46]. FISH mapping of the CCS1 *Avena-*700 sequence was restricted to centromeric domains in the *Avena* species here studied as formerly reported for similar sequences in *Triticum*, *Secale* and *Hordeum* chromosomes [47]. Furthermore, our FISH analysis in diploid and hexaploid *Avena* species also reinforces some phylogenetic aspects in *Avena* genus, due to its presence in all *A. strigosa* chromosomes but only in 2/3 of the chromosome complement from *A. sativa* and *A. sterilis.* This differential labeling suggests a distinct origin for C genome chromosomes as already proposed by Langdon and co-workrs [46] due to evidence on the absence of conserved retrotransposon centromeric sequences from several cereal species in the C genome putative progenitors of hexaploid oats such as *A. eriantha* and *A. ventricosa*. Moreover, the same authors also reported a reverse transcriptase probe derived from *A. strigosa*, the predicted A/D genome ancestor, which only labels A and D centromeric domains in *A. sativa* chromosomes, reinforcing therefore the suggested higher affinity of A and D genomes on hexaploid *Avena* (ACD) species [48].

*Avena* species/accessions discrimination was considerably increased by polymorphisms detected through the amplification of retrotransposons and microsatellites flanking sequences using IRAP and REMAP analysis [17]. Indeed, the accurate distinction of all *Avena* genotypes considered in this study was achieved through species and accession-specific bands identification, paving the way for the establishment of fast and simple molecular approaches to clearly identify *Avena* genotypes. Moreover, the dendogram constructed based on concatenated centromeric, IRAP and REMAP banding profiles undoubtedly allows a clear assessment of *Avena* diversity clustering all genotypes in two clades, one with *A. strigosa* and the other corresponding to hexaploid species, although separating *A. sativa* and *A. sterilis* accessions. Considering that REMAP technique was previously used to assess hexaploid oat species phylogenetic relationships [25] and proved to be related with oat important traits such as height [24] and cadmium accumulation [49] our work reinforces the high relevance of such molecular markers [50] also in the assessment of *Avena* biodiversity pool regarding both cultivated and wild relatives.

## 4. Materials and Methods

### 4.1. Plant Material

In this work we have used two Portuguese landraces of *A. strigosa* (2*n* = 14, AsAs); two Portuguese landraces and one line from Finland extensively used for comparative analysis of *A. sativa* (2*n* = 6*x* = AACCDD); and one wild Portuguese accession of *A. sterilis*. All lines used are described in detail in Table 3.

Seeds were germinated in petri dishes and three plants of each accession were maintained in soil pots at 16 h/light (25 °C) and 8 h/dark (18 °C). For DNA extraction fresh and healthy young leaves of 1 month-old plants were frozen and kept at −80 °C until further use. For cytogenetic analyses root tips were collected, cold treated for 24 h, fixed in ethanol/acetic acid (3:1 *v*/*v*) and stored at −20 °C until use.

**Table 3:** Description of *Avena* accessions analyzed.

Species	Genomic Constitution	Accession	Origin
*A. strigosa*	AsAs 2*n* = 14	5284/PRT005	Madeira Island, Portugal
		BC2531/PRT083	Elvas, Portugal
*A. sativa*	AACCDD 2*n* = 6*x* = 42	“Kyto”, Clav8250/USDA	Finland
		2424/PRT005	Madeira, Island Portugal
		“S. Eulália”/PRT083	Elvas, Portugal
*A. sterilis*	AACCDD 2*n* = 6*x* = 42	PI267989/USDA	Portugal

PRT005: EAN Germplasm Bank, Oeiras; PRT083—ENMP, Elvas; USDA: United States Department of Agriculture, Agricultural Research Service.

### 4.2. Genomic Analysis through PCR

DNA extractions were performed using cetyltrimethylammonium bromide CTAB method [51]. PCR analysis was performed using different primers designed for 45S rDNA internal transcribed spacers (ITS), rye subtelomeric sequence (pSc200, Accession Number Z50039) [26], cereal centromeric sequences (CCS1, Accession Number, U52217; D primer) [28], retrotransposon *Nikita* and microsatellites (CA)9G. Primers used are described in detail in Table 4. PCR reactions were performed in a total volume of 20 microliter with 1× PCR buffer, 1.5 mM MgCl_2_, 0.25 mM dNTP’s, 1 mM each primer, 0.5 U Taq polymerase, 50 ng DNA template. PCR amplification reactions of centromeric, subtelomeric and 45S rDNA ITS used the following program: 5 min 94 °C, 30 cycles of 45 s 94 °C, 45 s 60 °C (54 °C for subtelomeric sequence) and 1 min of 72 °C, final extension of 10 min at 72 °C. IRAP and REMAP were performed accordingly to [17]. PCR products were run on 1.7% agarose gels using as molecular weight marker 1 kb Plus DNA Ladder (Invitrogen, Carlsbad, CA, USA). Amplification products were detected with ethidium bromide and photographed using a Bio-Rad GEL DOC 2000 (Bio-Rad Laboratories, Inc., Hercules, CA, USA). Each PCR banding pattern was considered reproducible after being obtained in at least three technical replicates for each PCR experiment. For each sequence and each line studied at least three individual plants were analyzed. For 45S rDNA internal transcribed spacers pTa71 plasmid, a 9 kb fragment from *T. aestivum* containing the 18S-5.8S-25S rDNA and intergenic spacers cloned in pUC18 [52], was also used as control template. Similarly, for pSc200 subtelomeric sequence rye genomic DNA was used as control template.

**Table 4:** Primers used in PCR analysis.

Sequence	Sequence Type	Primers	Reference
45S ITS1	rDNA	Fow 5′-TCCGTAGGTGAACCTGCGG Rev 5′-GCTGCGTTCTTCATCGATGC	[29]
45S ITS2	rDNA	Fow 5′-GCATCGATGAAGAACGCAGC Rev 5′-TCCTCCGCTTATTGATATGC	[29]
pSc200	Telomeric	Fow 5′-TCTTTGATCACCGTTTCTTCG Rev 5′-CCCCACCCATGTATGGATAA	[31]
CCS1	Centromeric	5′-GGTGCCCGATCTTTCGATGAGA	[28]
*Nikita* LTR	Retrotransposon	5′-CGCTCCAGCGGTACTGCC	[53]
(CA)9G	Microsatellite	5′-CACACACACACACACACAG	-

Selected bands were gel isolated, purified using High Pure PCR Product Purification Kit (Roche, Basel, Switzerland), cloned using TOPO TA Cloning Kit in pCR2.1 vector (Invitrogen, Carlsbad, CA, USA) and sequenced. At least three distinct clone per sequence/line were analyzed.

### 4.3. Sequence Similarity Analysis

Sequences from isolated bands were verified and aligned using BioEdit version 7.1.3.0 [54] and analyzed along with sequences obtained from DDBJ/EMBL/GenBank International Nucleotide Sequence Database. For sequence similarity analyses Clustal W2 [30] was used.

Inter Retrotransposons Amplified Polymorphism (IRAP), Retrotransposons Microsatellite Amplified Polymorphism (REMAP) and centromeric sequences PCR banding profiles obtained were transformed in binary matrices, where 1 corresponds to band presence and 0 to absence. NTSYSpc software [55] was used to infer genetic similarity between all accessions and species using Dice coefficient [36]. Dendrograms were obtained through UPGMA (Unweighted Pair Group Method with Arithmetic Mean) method for a concatenated binary of CCS1, IRAP and REMAP profiles obtained using Concatenator Software [56]. Cophenetic correlation coefficient (*r*) was estimated to verify the adjustment between similarity matrix and respective dendrogram-derived matrix. The determination of confidence limits by bootstrap analysis was done using the WINBOOT software.

### 4.4. Cytogenetics Analysis through Fluorescent in Situ Hybridization (FISH)

FISH analysis was performed as described in [45] in meristematic cells spreads obtained from root tips fixed in ethanol/acetic acid (3:1) and digested with pectinase/cellulose. Pre-hybridization treatments to remove proteins and RNA were performed with 6.67 µg/mL pepsin and 100 ng/µL RNAse, respectively. pTa71 probe, a plasmid that includes a 9 kb EcoRI fragment of the rDNA unit from wheat (*Triticum aestivum*) [52] was labeled with digoxigenin-dUTP or biotin-dUTP (Roche, Basel, Switzerland) through Nick Translation. Centromeric sequence (Accession Number KM948610) was amplified from *A. sativa* and labeled with biotin-dUTP (Roche) through PCR using CCS1 primers. The subtelomeric probe pSc200 was amplified from *A. sativa* DNA and labeled with digoxigenin-dUTP through PCR. For each slide 50 ng of pTa71, 100 ng of centromeric sequence and 100 ng of pSc200 were diluted in a hybridization mixture composed by 50% formamide, 2× SSC (saline-sodium citrate buffer), 0.17% SDS (sodium dodecyl sulfate), 1 µg/µL salmon sperm, 10% dextrane sulphate (stringency of 77%). Post-hybridization washes were done with a stringency of 85% and bovine serum albumin 5% (*w*/*v*) was used as blocking reagent before probe detection with 2 µg/mL of Anti-digoxigenin Fluorescein (FITC) and 5 µg/mL Streptavidin-Cy3. Cells were counterstained with 4′,6-diamidino-2-phenylindole hydrochloride (DAPI) in Citifluor antifade mounting medium (AF1; Agar Scientific, Stansted, Essex, UK). Samples were examined using a Zeiss AxioImager Z1 epifluorescence microscope. Images were obtained using a Zeiss AxioCam HRm digital camera (Zeiss, Oberkochen, Germany) and the selected ones were processed using Photoshop (Adobe Systems, San Jose, CA, USA). The described FISH signals illustrated in Figure 1, Figure 2 and Figure 5 were observed in at least 10 metaphase or prometaphase cells. 45S rDNA labeling was analyzed through the comparison of FISH signals sizes within each cell, considering that all chromosomes in the same cell assume a similar condensation level.

## 5. Conclusions

Genomic diversity and molecular discrimination of the most relevant crop and weed *Avena* species from Portugal were assessed through molecular and cytological evaluation of distinct repetitive sequences. FISH analysis of 45S rDNA revealed inter and intra-specific variability though the ITS high similarity observed. pSc200-like sequences were identified and revealed high homology in the distinct genotypes analyzed. Conversely, the analysis of centromeric banding patterns along with profiles produced by retrotransposons and microsatellites targeting methodologies (IRAP and REMAP) allowed an accurate assessment of the variability, providing novel prospective molecular markers for future oat breeding programs.

## Supplementary Materials

Supplementary materials can be found at http://www.mdpi.com/1422-0067/17/2/203/s1.
