# Supplementary material for: Use of Repetitive Sequences for Molecular and Cytogenetic Characterization of Avena Species from Portugal
Source: Int J Mol Sci. 2016 Feb 4;17(2):203. doi: 10.3390/ijms17020203 (PMC4783937; doi:10.3390/ijms17020203)
Supplement: Supplementary file 1 [file ijms-17-00203-s001.pdf]

Supplementary Material: Use of Repetitive Sequences for Molecular and Cytogenetic Characterization of *Avena* Species from Portugal

Diana Tomás, Joana Rodrigues, Ana Varela, Maria Manuela Veloso, Wanda Viegas and Manuela Silva

```
                                          10         20         30         40         50         60         70         80         90
                                  ....|....| ....|....| ....|....| ....|....| ....|....| ....|....| ....|....| ....|....| ....|....|
pSc200_Avena_strigosa_Mad         CCCCACCCAT GTATGGATAA AATAGGGTGT TAGGCCAACA CTCGCGAAGA AACGGTGATC AAAGAAAATA CAAAAATCAA CCTAGAGTCC
pSc200_Avena_strigosa_Elv         .......... .......... .......... .......... .......... .......... .......... .......... ..........
pSc200_Avena_sativa_Kyto          .......... .......... .......... .......... .......... .......... .......... .......... ..........
pSc200_Avena_sativa_Madeira       .......... .......... .......... .......... .......... .......... .......... .......... ..........
pSc200_Avena_sativa_S.Eulalia     .......... .......... .......... .......... .......... .......... .......... .......... ..........
pSc200_Avena_sterilis_Pt          .......... .......... .......... .......... .......... .......... .......... .......... ..........
pSc200_Secale_cereale_(Z50039)    .......... .......... .......... .......... .......... .......... .......... .......... ..........

                                         100        110        120        130        140        150        160        170        180
                                  ....|....| ....|....| ....|....| ....|....| ....|....| ....|....| ....|....| ....|....| ....|....|
pSc200_Avena_strigosa_Mad         GAATTGATCG AGACTCTCAA AGCAAGTGAG A--ACAAGCA ACGATTTCGT CGTTTTCATA GGCGTTGGAA AGCTATTGGG GAATTGAGGT
pSc200_Avena_strigosa_Elv         .......... .......... .......... .--....... .......... .......... .......... .......... ..........
pSc200_Avena_sativa_Kyto          .......... .......... .......... .--....... .......... .......... .......... .......... ..........
pSc200_Avena_sativa_Madeira       .......... .......... .....A.... .--....... .......... .......... .......... .......... ..........
pSc200_Avena_sativa_S.Eulalia     .......... .......... .......... .--....... .......... .......... .......... .......... ..........
pSc200_Avena_sterilis_Pt          .......... .......... .......... .--....... .......... .......... .......... .......... ..........
pSc200_Secale_cereale_(Z50039)    .......... .......... .......... .GA....... .......... .......... .......... .......... ..........

                                         190        200        210        220        230        240        250        260        270
                                  ....|....| ....|....| ....|....| ....|....| ....|....| ....|....| ....|....| ....|....| ....|....|
pSc200_Avena_strigosa_Mad         GATAGGACTT CTAGTTTTTA TGATCTGGAG GATTTTACAT ACGTGCACAC ACAGGGTCAC GTGCGTGGAA AATTCTGGCC CAAAAAGTGT
pSc200_Avena_strigosa_Elv         .......... .......... .......... .......... .......... .....A.... .......... .......... ..........
pSc200_Avena_sativa_Kyto          .......... .......... .......... .......... .......... .....A.... .......... .......... ..........
pSc200_Avena_sativa_Madeira       .......... .......... .......... .......... .......... .....A.... .......... .......... ..........
pSc200_Avena_sativa_S.Eulalia     .......... .......... .......... .......... .......... .....A.... .......... .......... ..........
pSc200_Avena_sterilis_Pt          .......... .......... .......... .......... .......... .....A.... .......... .......... ..........
pSc200_Secale_cereale_(Z50039)    .......... .......... .......... .......... .......... .....A.... .......... .......... ..........

                                         280        290        300        310        320        330        340        350        360
                                  ....|....| ....|....| ....|....| ....|....| ....|....| ....|....| ....|....| ....|....| ....|....|
pSc200_Avena_strigosa_Mad         CGTCTTGGCC CTCAAAAATG GCCTGCACGA TTGGAAAAAA TGAAAGTTAT CGCAATTACA GCTCAAATTT TGATGAGCCG GCATATCACT
pSc200_Avena_strigosa_Elv         .......... .......... .......... .......... .......... .......... .......... .......... ..........
pSc200_Avena_sativa_Kyto          .......... .......... .......... .......... .......... .......... .......... .......... ..........
pSc200_Avena_sativa_Madeira       .......... .......... .......... .......... .......... .......... .......... .......... ..........
pSc200_Avena_sativa_S.Eulalia     .......... .......... .......... .......... .......... .......... .......... .......... ..........
pSc200_Avena_sterilis_Pt          .......... .......... .......... .......... .......... .......... .......... .......... ..........
pSc200_Secale_cereale_(Z50039)    .......... .......... .......... .......... .......... .......... .......... .......... ..........

                                          370        380        390        400        410        420        430        440
                                   ....|....| ....|....| ....|....| ....|....| ....|....| ....|....| ....|....| ....|....| ....|.
pSc200_Avena_strigosa_Mad          TTGGTGTTCA GGTCCTTGGT GCCCCACCCA TGTATGGATA CAATAGGGTG TTAGGCCAAC TCTCGCGAAG AAACGGTGAT CAAAGA
pSc200_Avena_strigosa_Elv          .......... .......... .......... .......... A......... .......... .......... .......... ......
pSc200_Avena_sativa_Kyto           .......... .......... .......... .......... A......... .......... A......... .......... ......
pSc200_Avena_sativa_Madeira        .......... .......... .......... .......... A......... .......... .......... .......... ......
pSc200_Avena_sativa_S.Eulalia      ......C... .......... .......... .......... .......... .......... .......... ........G. ......
pSc200_Avena_sterilis_Pt           .......... .......... .......... .......... A......... .......... A......... .......... ......
pSc200_Secale_cereale_(Z50039)     .......... .......... .......... .......... .......... .......... .......... .......... ......
```

Figure S1. Alignment of pSc200-like sequences obtained from *A. strigosa* ("Madeira Island" and "Elvas"), *A. sativa* ("Kyto", "Madeira Island" and "S. Eulália"), *A. sterilis* and *S. cereale* subtelomeric sequence (Accession Number Z50039).

```
      10         20         30         40         50         60         70         80         90
                               ....|....| ....|....| ....|....| ....|....| ....|....| ....|....| ....|....| ....|....| ....|....|
Centrom_A_strigosa_Elv_c1      TGGTGCCCGA TCTTTCGATG AGATGGTGAT AGTCGATTTG GTGGAGTTCG GGCTTGACGA TCCGACTACA CGTGCGAGCA TGAGCGCCAA
Centrom_A_strigosa_Elv_c2      .......... .......... ...A...... .......... .......... .......... .......... .A........ C.........
Centrom_A_strigosa_Elv_c3      .......... .......... .......... .......... .......... ..A....... .......T.. .......... C........T
Centrom_A_sativa_S.Eul_c1      .......... .......... .......... .......... ...A...... ..T....... .......... T......... C.........
Centrom_A_sativa_S.Eul_c2      .......... .......... .....A.A.. G......... .......... .......T.. .....T.... .A....T... C.........
Centrom_A_sativa_S.Eul_c3      .......... .......... ......C... .A........ .......... .......... .......... .......... C.........
Centrom_A_sterilis_Pt_c1       .......... .......... ......C... .A........ .......... .......... .......... .......... C.........
Centrom_A_sterilis_Pt_c2       .......... .......... .......... .......... .......... .......... .......... .......... C.........
Centrom_A_sterilis_Pt_c3       .......... .......... .......... .A........ ........A. .......... .......... .......... C.........
Centrom_A_strigosa_Mad         .......... .......... ......C... .A........ .......... .......... .......... .......... C.........
Centrom_A_sativa_Kyto          .......... .......... .......... .......... ......---- ---------- --------T. ........A. C.........

                                      100        110        120        130        140        150        160        170        180
                               ....|....| ....|....| ....|....| ....|....| ....|....| ....|....| ....|....| ....|....| ....|....|
Centrom_A_strigosa_Elv_c1      TGCAATCGCT AGGACAATCT CCTGGAGGTT ACCGATCCTG TGGGAACACG ATCAGCCTGA CCAGCGAGGG TCTTAATTCC TATCTGAAAT
Centrom_A_strigosa_Elv_c2      .......... ...C...... .......... .T........ C..A...... .......... .......... .......... ..C.......
Centrom_A_strigosa_Elv_c3      .......... ...C...... .......... .......... .......... .......... ...A...... .......... ..C.......
Centrom_A_sativa_S.Eul_c1      .......... ...C...... .......... .......... C......... ........A. ...A...... .......... ..C.......
Centrom_A_sativa_S.Eul_c2      ..A....... ...C...... .......... ..T....... C..T...... .......... .....A.... .......... ..C.......
Centrom_A_sativa_S.Eul_c3      .......... ...C...... ......A... .......T.. C.T....... .......... .......... .......... ..C.......
Centrom_A_sterilis_Pt_c1       .......... ...C...... .......... .......... C.T....... G......... .......... .......... ..C.......
Centrom_A_sterilis_Pt_c2       ..G....... ...C...... ...A...... .......... C......... .......... .......... .......... ..C.......
Centrom_A_sterilis_Pt_c3       .......... ...C...... .......... .......... C......... .......... .......... .........G ..C.......
Centrom_A_strigosa_Mad         .......... ...C...... ...A...... ...A...... C......... .......... .......... .......... ..C.......
Centrom_A_sativa_Kyto          .......... ...C...... .......... .......... CA........ .......... .......... .......... ..C.......

                                      190        200        210        220        230        240        250        260        270
                               ....|....| ....|....| ....|....| ....|....| ....|....| ....|....| ....|....| ....|....| ....|....|
Centrom_A_strigosa_Elv_c1      CGAGAACAAG TAAGAACTAA TATTGCAATT AAGATATTGC GGATTAAGAT GAAAGCTTTA TTGAAA-AGG TGGGATCCTA TATAGGTGCG
Centrom_A_strigosa_Elv_c2      .......... .......C.. .........C G......... .T.......G .......... .....-.... .........G A.........
Centrom_A_strigosa_Elv_c3      ........G. .......... .........C .........T .......... .......... .....-.... .........G ..........
Centrom_A_sativa_S.Eul_c1      ...T...... .......C.. .........C .........T .......... .......... .....A.... ..C......G ......C.T.
Centrom_A_sativa_S.Eul_c2      .......... .......... .........C .......... A......... .......... .....-.... .......A.G ..........
Centrom_A_sativa_S.Eul_c3      .......... .......C.. .........C .......... .........G .......... .....G.... .........G A.........
Centrom_A_sterilis_Pt_c1       .......... .......C.. .........C .......... .........G .......... .....G.... .........G A.........
Centrom_A_sterilis_Pt_c2       .......... .......... .........C .........T .......... .......... .....-.... .........G ..........
Centrom_A_sterilis_Pt_c3       .......... .......C.. .........C ..T....... .........G .......... .....-.... .........G ..........
Centrom_A_strigosa_Mad         .......... .......C.. .........C .......... .........G .......... .....-.... .........G ..........
Centrom_A_sativa_Kyto          .......... .......... .........A .......... .......... .....A.... .....-.... .........G ..........
280        290        300        310        320        330        340        350        360
```

Figure S2. *Cont.*

```
                                ....|....| ....|....| ....|....| ....|....| ....|....| ....|....| ....|....| ....|....| ....|....|
Centrom A_strigosa_Elv c1       AAGTACTAGA GGCCTTTGCG ATGCTCGAGC CTTGGCAAAG TTGCTATGAT GGCTAACTTA ATGTCTAATC AAAATCCGAG TCTATGGTGA
Centrom A_strigosa_Elv c2       ......A... .....C.... .C........ ......G... ......C... .......... ........A. .......... ...TG.....
Centrom A_strigosa_Elv c3       ......A... .....C.... .C........ .....AG... .......... .......... T.......A. .......... .A........
Centrom_A_sativa_S.Eul c1       ......A... .....C.... .CA....... ......G... .......... .......... ........A. .......... ....C.....
Centrom_A_sativa_S.Eul c2       ......A... A....C.... .C........ .......... .......... .......... ........A. .......... ..........
Centrom_A_sativa_S.Eul c3       ......A... .....C.... .C........ ......G... ....G.CT.. .......... ...C....A. .......... ....GA.GTT
Centrom_A_sterilis_Pt c1        ......A... .....C.... .C........ ......G... ....G.CT.G .......... ........A. .......... ....GA.GTT
Centrom_A_sterilis_Pt c2        ......A... .....A.... .....T.... ......G... C......... .........T ........A. .......... .G..AAA...
Centrom_A_sterilis_Pt c3        ......A... .....C.... .C........ ......G... ......C... ...A...... ........A. .......C.. ....G.....
Centrom_A_strigosa_Mad          ......A... .....C.... .C........ ....A.G... ......C... ........-. ........AT .......A.. ....G.....
Centrom_A_sativa_Kyto           ......A... .....C..T. .C........ ......G... .......... .......... ........A. .......... ..........

                                       370        380        390        400        410        420        430        440        450
                                ....|....| ....|....| ....|....| ....|....| ....|....| ....|....| ....|....| ....|....| ....|....|
Centrom A_strigosa_Elv c1       TGGCTAAGGC GAGTATTTAA GGAGGAAAAG GTAGGGTTTC AGCCAGCTAT AGAAGTGGTG GCCGAATCTA ACCCTAGAAG TCCTTTTCTC
Centrom A_strigosa_Elv c2       A.T...T..G .......... ..GC...... ..G....... G......C.. G......A.. ........C. .......... ........C.
Centrom A_strigosa_Elv c3       C......... .......... .......... .......... .....A.C.. .......... ........C. .......... ..........
Centrom_A_sativa_S.Eul c1       C.....T..G .......... .......... ..G....... G......C.. .........T ........C. .......... ..........
Centrom_A_sativa_S.Eul c2       ..A....... .......... .......... .......... GT.....C.. .......... ........C. .T.G...... ..........
Centrom_A_sativa_S.Eul c3       C.....T..G .C........ ..GC...... ..G....... G.....TC.. G......... ........C. ......A... ........C.
Centrom_A_sterilis_Pt c1        C.....T..G .C........ ..GC...... ..G....... G......C.. G......... ...T....C. ......A... ........C.
Centrom_A_sterilis_Pt c2        C.A...T..G ..C....... .......... ..G....... .T...T.C.. ...G..A... ........C. .........A .G........
Centrom_A_sterilis_Pt c3        C.A...T..G .......... .......... ..G....... G......C.. G......... ...A....C. .......... ........C.
Centrom_A_strigosa_Mad          C.....T..G .......... .......... ..G....... G......C.. G......... ........C. ...G...... .T......C.
Centrom_A_sativa_Kyto           C......... .......... .......... .......... G......C.. .......... ........C. .......... ..........

                                       460        470        480        490        500        510        520        530        540
                                ....|....| ....|....| ....|....| ....|....| ....|....| ....|....| ....|....| ....|....| ....|....|
Centrom A_strigosa_Elv c1       CTCAAATATG GACTCTAAAA TGTGGCTATT GAAAATCCTG CAAAAATTAC ATGGGCCTGG CCCAAAATAA AGGTGTCGCG GCACCATATT
Centrom A_strigosa_Elv c2       ........C. .......... A.......A. T......... .G........ .......... .......... .........A ..........
Centrom A_strigosa_Elv c3       ........CA .......C.. A......... T......... TG........ .....T.... .......... .......A.A A.........
Centrom_A_sativa_S.Eul c1       ........C. .......... A.......A. T......... .G........ .......... .......... .........A ..........
Centrom_A_sativa_S.Eul c2       ........C. .......... A......... T......... .G.......A .......... .......... .........A ..........
Centrom_A_sativa_S.Eul c3       ........CA .......... A.......A. T......... .G........ .......... ......T... .........A ..........
Centrom_A_sterilis_Pt c1        ........C. .......... A.......A. T......... .G........ .......... .......... .........A ..........
Centrom_A_sterilis_Pt c2        ........C. .......... A.......A. T......T.. TG.....A.. .......... .......... .........A ..........
Centrom_A_sterilis_Pt c3        ........C. .......... A.......A. T......... .G........ .......... ....C..... .........A ..........
Centrom_A_strigosa_Mad          ........C. .......... A......... T...T....A .G...G.... .........A .......... ........TA A.........
Centrom_A_sativa_Kyto           ........CA .......... A......... T......... TG........ ..A....... .......... .......... ..........
                                       550        560        570        580        590        600        610        620        630

                                ....|....| ....|....| ....|....| ....|....| ....|....| ....|....| ....|....| ....|....| ....|....|

Centrom A_strigosa_Elv c1       AAGCTATGGA CGAATTTGTG AAGTATTAAT CTTGAACATT TCGTCCATGC CTTCTTTCTT CATTATGGTG GCCTCAAAGT TCTGAAATCT
Centrom A_strigosa_Elv c2       .......... ......C... .......T.. ..C...T... .......A.. .......... ...-...... .......... ..........
Centrom A_strigosa_Elv c3       .......... .......... .......... ......T... .......A.A .......... .......... .......... ..........
Centrom_A_sativa_S.Eul c1       .......... .......... ........G. ......T... .T.....A.. ...T...... .......... .......... ..........
Centrom_A_sativa_S.Eul c2       .........T ........C. .......... ......T... .......A.. .......... .......... .......... ..........
Centrom_A_sativa_S.Eul c3       ......A... ......C..A .......G.. ......T... .......A.. .......... .......... .......... ..........
Centrom_A_sterilis_Pt c1        .T........ ......C..A .......G.. ......T... .T.....A.. .......... .......... .......... ..........
Centrom_A_sterilis_Pt c2        .......... .......... ....GC.... ......T... .T.....A.. ....AA.A.C .......... ..T....... ........A.
Centrom_A_sterilis_Pt c3        .......... ......CA.. .......... ......T... .......A.. .......... .......... .......... ..........
Centrom_A_strigosa_Mad          .......... ......C... .......... ......T... .......A.. .......A.. .......... .......... G.........
Centrom_A_sativa_Kyto           .......... .......... .......... ......T... .......A.. .......... .......... .......... ..........

                                       640        650        660        670        680        690        700        710        720
                                ....|....| ....|....| ....|....| ....|....| ....|....| ....|....| ....|....| ....|....| ....|....|
Centrom A_strigosa_Elv c1       TCATCTGTGG CCTTCTCTTG GATCATCACA TGCTTG---- ---------G CATGACC-AT GATCCAATGC TTGTCCCTCT CATCGAAAGA
Centrom A_strigosa_Elv c2       .....CA... ........C. .......... C....TCTGC CATCCCCAT. ....GG.C.. .C........ .....A.... ..........
Centrom A_strigosa_Elv c3       .......... .......... .......... ......CTGC CATCTCCAT. .......-.. .CA....... .......... ..........
Centrom_A_sativa_S.Eul c1       .......... .T........ A......... ......CTGC CATCTCCAT. ....G..-.. .C........ .......... ..........
Centrom_A_sativa_S.Eul c2       .......... .......... A......... ......CTGC CATCTCCAT. .......-.. .C........ .......... ..........
Centrom_A_sativa_S.Eul c3       .......... .......... .T........ ......CTGC CATCTCCAT. ....G..-.. .C........ .......... ..........
Centrom_A_sterilis_Pt c1        .....C.... .......... .......... ......CTGC CATCTCCAT. ....G..C.. .C........ .......... ..........
Centrom_A_sterilis_Pt c2        C..CTC.... .G.C.....T A...C..... ......CTGC CATATCCAT. .....T.-.. .C........ ......T... ..........
Centrom_A_sterilis_Pt c3        .......... .......... .......... ......CTGC CAT------- ---------- .C........ .-........ ..........
Centrom_A_strigosa_Mad          C....C.... .......... .......... ......CTGC CATCTCCAT. ....G..-.. .C.......T .......... ..........
Centrom_A_sativa_Kyto           .....C...T ......A... .......... ......CTTC CATCTCCAT. .......-.. .C......A. ..A..T.... ..........

                                       730
                                ....|....|
Centrom A_strigosa_Elv c1       TCGGGCACCA
Centrom A_strigosa_Elv c2       ..........
Centrom A_strigosa_Elv c3       ..........
Centrom_A_sativa_S.Eul c1       ..........
Centrom_A_sativa_S.Eul c2       ..........
Centrom_A_sativa_S.Eul c3       ..........
Centrom_A_sterilis_Pt c1        ..........
Centrom_A_sterilis_Pt c2        ..........
Centrom_A_sterilis_Pt c3        ..........
Centrom_A_strigosa_Mad          ..........
Centrom_A_sativa_Kyto           ..........
```

Figure S2. Alignment of Centromeric Sequences obtained from *A. strigosa* ("Madeira Island" and "Elvas"), *A. sativa* ("Kyto" and S. Eulália") and *A. sterilis*.

Table S1. GenBank Accession Numbers of pSc200 and Centromeric Sequences.

Sequence	Accession Numbers
pSc200_Avena_strigosa_Mad	KM948601
pSc200_Avena_strigosa_Elv	KM948602
pSc200_Avena_sativa_Kyto	KM948603
pSc200_Avena_sativa_Madeira	KM948604
pSc200_Avena_sativa_S.Eulalia	KM948605
pSc200_Avena_sterilis_Pt	KM948606
Centromeric_ Avena_strigosa_Elv _clone1	KM948607
Centromeric_ Avena_strigosa_Elv _clone2	KM948608
Centromeric_ Avena_strigosa_Elv_ clone3	KM948609
Centromeric_ Avena_sativa_S.Eulalia _clone1	KM948610
Centromeric_ Avena_sativa_S.Eulalia _clone2	KM948611
Centromeric_ Avena_sativa_S.Eulalia _clone3	KM948612
Centromeric_ Avena_sterilis_Pt _clone1	KM948613
Centromeric_ Avena_sterilis_Pt _clone2	KM948614
Centromeric_ Avena_sterilis_Pt _clone3	KM948615
Centromeric_ Avena_strigosa_Mad	KM948616
Centromeric_ Avena_sativa_Kyto	KM948617
